# One-year seropositivity after COVID-19 infection: A study from a developing country

**DOI:** 10.1097/MD.0000000000047902

**Published:** 2026-03-06

**Authors:** Nasr Alrabadi, Haneen Obeidat, Razan Haddad, Osama Alzoubi, Daher Al-rabadi, Saied Jaradat, Maryam K. El-zubi, Rania Harati, Karem H. Alzoubi, Ibrahim Al-faouri

**Affiliations:** a Department of Pharmacology, Faculty of Medicine, Jordan University of Science and Technology, Irbid, Jordan; b Department of Medical Laboratory Sciences, Faculty of Applied Medical Sciences, Jordan University of Science and Technology, Irbid, Jordan; c Department of Pharmaceutical Sciences, Faculty of Pharmacy, Jadara University, Irbid, Jordan; d Division of Rheumatology, Department of Medicine, University of Illinois at Chicago, IL; e Department of Community and Mental Health Nursing, Faculty of Nursing, Jordan University of Science and Technology, Irbid, Jordan; f Princess Haya Biotechnology Center, Jordan University of Science and Technology, Irbid, Jordan; g Department of Pharmacy Practice, College of Pharmacy, Gulf Medical University, Ajman, United Arab Emirates; h Department of Pharmacy Practice and Pharmacotherapeutics, University of Sharjah, Sharjah, United Arab Emirates; i Research Institute for Medical and Health Sciences, University of Sharjah, Sharjah, United Arab Emirates; j Department of Pharmacy Practice and Pharmacotherapeutics, College of Pharmacy, University of Sharjah, Sharjah, United Arab Emirates; k Department of Clinical Pharmacy, Faculty of Pharmacy, Jordan University of Science and Technology, Irbid, Jordan; l College of Nursing, RAK Medical and Health Sciences University, Ras Al Khaimah, United Arab Emirates.

**Keywords:** antibodies, COVID-19 disease, developing country, 1 year postinfection, seropositivity, spike protein

## Abstract

Understanding the duration of the humoral immune response after coronavirus disease 2019 infection is critical for assessing vaccination strategies. This study aimed to evaluate the persistence of severe acute respiratory syndrome coronavirus 2 (SARS-CoV-2) antibodies in patients from Northern Jordan 1 year after infection. A descriptive cross-sectional study was conducted at King Abdullah University Hospital in Irbid, Jordan, from January to March 2021. Participants who were infected with the early SARS-CoV-2 variants circulating in Jordan (B.1, B.1.1, and B.1.1.312) prior to the emergence of the Alpha variant and unvaccinated were tested for antibodies against recombinant spike (S) and nucleocapsid (N) proteins using the quantitative Elecsys Anti-SARS-CoV-2 S and the qualitative Elecsys Anti-SARS-CoV-2 assays, respectively (Roche Diagnostics GmbH, Mannheim). Data were analyzed using Statistical Package for Social Sciences, and chi-square tests were applied to identify associations. Among 122 participants (62 males, 60 females), more than 50% of the patients retained detectable antibodies 1 year postinfection. Spike protein antibodies showed a more consistent long-term presence compared to nucleocapsid antibodies. Blood group O individuals showed the lowest seropositivity rates (*P* = .06). Other variables, including age, gender, comorbidities, smoking status, and symptom severity, did not show a significant correlation with antibody persistence. SARS-CoV-2 antibodies persist in more than 50% of patients for at least 1 year postinfection, particularly against the spike protein. These findings support the potential for long-term immune protection and may inform vaccination policies, particularly in resource-limited settings.

## 1. Introduction

The coronavirus disease 2019 (COVID-19) pandemic, caused by the severe acute respiratory syndrome coronavirus 2 (SARS-COV-2), has posed unprecedented challenges globally. The virus, a positive-sense single-stranded RNA pathogen, spreads through airborne transmission and close contact,^[1–3]^ causing a wide range of symptoms from mild respiratory illness with fever, cough, headache, fatigue, breathing difficulties, and loss of taste and smell, to more severe complications such as respiratory failure.^[4–6]^ The first known case was identified in December 2019 in Wuhan, China, and since then, the disease has spread rapidly worldwide.

Since the World Health Organization declared COVID-19 a pandemic on March 11, 2020, efforts have been made to control its spread through public health interventions and vaccination campaigns.^[7,8]^ Scientists have employed measures such as social distancing, mask-wearing, frequent hand-washing, and even nationwide quarantines to reduce the impact of the virus.^[9–12]^ Despite these efforts, the virus continued circulating, leading to persistent concerns regarding reinfection and long-term immunity.

A key aspect of pandemic management is understanding the duration of immune protection following natural infection. This helps determine the need for and timing of booster vaccinations, as well as prioritization of vaccinations to ensure that high-risk or nonimmunized individuals and health workers receive doses first, especially when resources are limited.^[13]^

Seropositivity, the presence of detectable antibodies, indicates prior exposure and potential immunity, and is often used as a marker of immune protection. Previous studies suggest that antibody levels decline over time, but the exact duration and factors influencing seropositivity remain unclear, particularly in developing countries. Like any virus, seropositivity develops in individuals exposed to COVID-19 or vaccinated against it, with antibody titers expected to wane over time. Previous literature has demonstrated that antibody titers vary depending on many factors, including the duration of the disease and individual characteristics such as smoking history.^[13]^ Understanding these factors and the duration for which patients can retain antibodies after infection is critical for informing public health strategies and optimizing vaccination policies, particularly in resource-limited settings.

Among the primary targets of serological studies, the spike (S) and nucleocapsid (N) proteins of SARS-CoV-2 play distinct roles in immune response and viral pathogenesis. The S protein is responsible for viral entry into host cells through interaction with the angiotensin-converting enzyme 2 receptor, making it the primary target of neutralizing antibodies and vaccine-induced immunity. In contrast, the N protein, which is highly abundant within the virus, serves as a major immunogenic component in natural infections and is commonly used for diagnostic testing.^[14]^ Studies suggest that antibodies against the S protein persist for longer periods compared to those against the N protein.^[15]^ Therefore, evaluating antibody responses to the S and N proteins is essential when assessing long-term immunity.

This study aimed to determine the percentage of seropositivity among individuals living in a developing country by analyzing antibodies against the S and N proteins and their changes over time. Additionally, the study aimed to investigate correlations between antibody titers and patient characteristics, including gender, blood type, comorbidities, smoking history, and the severity of symptoms during illness.

## 2. Materials and methods

### 2.1. Study design

This descriptive cross-sectional study was conducted at King Abdullah University Hospital in Irbid, Jordan, between January and March 2021. The study protocol was approved by the Institutional Review Board of Jordan University of Science and Technology (Approval number: 130/136/2020). Written informed consents were obtained from all study participants.

### 2.2. Participants and data collection

**Figure 1.**
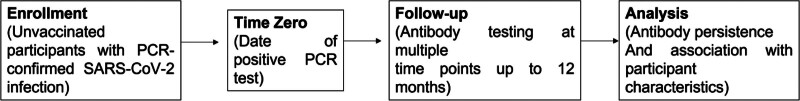
Study workflow. Enrollment included unvaccinated participants with PCR‐confirmed SARS‐CoV‐2 infection. The date of the positive PCR test was designated as time zero. Participants were followed up with antibody testing at multiple time points up to 12 months post‐infection. Data were then analyzed to assess antibody persistence and its association with patient characteristics. PCR = polymerase chain reaction, SARS-CoV-2 = severe acute respiratory syndrome coronavirus 2.

Participants in the study included unvaccinated patients visiting King Abdullah University Hospital who were infected with the early SARS-CoV-2 variants circulating in Jordan (B.1, B.1.1, and B.1.1.312) prior to the emergence of the Alpha variant. Indeed, a national genomic sequencing of 579 SARS-CoV-2 samples collected in Jordan between March and December 2020 showed that the predominant lineages were B.1 and B.1.1 during March–May 2020, followed by B.1.1.312 as the dominant lineage in August 2020. The Alpha variant (B.1.1.7) was first detected in early 2021 and triggered the second wave of the epidemic, with a surge in cases by March 2021. Our study participants were infected during the early epidemic period (March–December 2020), that is, prior to the widespread circulation of the Alpha variant. At the time of recruitment, vaccination in Jordan was limited to selected high-risk groups through the government program, which commenced in January 2021. Our study included all eligible and consenting participants within the recruitment window. The sample size was determined by the maximum feasible number of cases available during the period prior to the widespread rollout of COVID-19 vaccination in Jordan and before the emergence of new variants. This approach ensured that participants were representative of infections with the early circulating SARS-CoV-2 lineages, while also minimizing the risk of reinfection influencing the results. Vaccination status of participants was verified through Ministry of Health records and confirmed by participant self-report; only unvaccinated individuals were eligible to participate. COVID-19 infection was confirmed in all participants by a positive RT-PCR test, which was designated as time zero for the duration of the observation period. No restrictions were placed on age, gender, or ethnicity. An informed written consent was obtained from all participants in the study, and participants completed questionnaires covering demographic data, specifically age, gender, blood type, medical history, presence of comorbidities (diabetes mellitus, hypertension, asthma, hypothyroidism, encephalitis), smoking status and symptoms severity during time of infection. Patients with known immunocompromising conditions, such as human immunodeficiency virus (HIV) infection, active malignancy, or a history of organ transplantation, were excluded from participation. The clinical characteristics of included participants are summarized in Table 1. Serum samples were collected and tested for SARS-CoV-2 antibodies against S and N proteins using the quantitative Elecsys Anti-SARS-CoV-2 S and the qualitative Elecsys Anti-SARS-CoV-2 assays, respectively (Roche Diagnostics GmbH, Mannheim, Germany) according to the manufacturer’s procedure. The Cobas 6000 was used for incubation. The different participants were tested for antibodies over a 12-month period, each with a varying number of months postinfection. The study workflow is illustrated in Figure 1.

**Table 1:** Demographic and clinical characteristics of study participants and their SARS-CoV-2 seropositivity data.

	Total		Nucleocapsid (N) protein antibody					Spike (S) protein antibody				
			No		Yes		*P* value	No		Yes		*P* value
	Count	%	Count	%	Count	%		Count	%	Count	%	
Age (mean)	37.45		36.67		37.83		.705	42.64		36.32		.089
Gender												
Male	62	50.8	16	41.0	46	55.4	.138	11	50.0	51	51.0	.932
Female	60	49.2	23	59.0	37	44.6		11	50.0	49	49.0	
Blood.type												
A	33	33.0	5	17.9	28	38.9	.006	4	22.2	29	35.4	.106
B	30	30.0	5	17.9	25	34.7		3	16.7	27	32.9	
O	35	35.0	17	60.7	18	25.0		10	55.6	25	30.5	
AB	2	2.0	1	3.6	1	1.4		1	5.6	1	1.2	
Rh factor												
Positive	94	94.0	25	89.3	69	95.8	.216	16	88.9	78	95.1	.313
Negative	6	6.0	3	10.7	3	4.2		2	11.1	4	4.9	
Comorbidities												
No	30	24.6	32	82.1	60	72.3	.243	17	77.3	75	75.0	.823
Yes	14	11.5	7	17.9	23	27.7		5	22.7	25	25.0	
DM												
No	19	15.6	33	84.6	75	90.4	.353	18	81.8	90	90.0	.276
Yes	2	1.6	6	15.4	8	9.6		4	18.2	10	10.0	
HTN												
No	7	5.7	35	89.7	68	81.9	.267	19	86.4	84	84.0	.782
Yes	38	31.1	4	10.3	15	18.1		3	13.6	16	16.0	
Asthma												
No	17	13.9	39	100.0	81	97.6	.328	22	100.0	98	98.0	.504
Yes	105	86.1	0	0.0	2	2.4		0	0.0	2	2.0	
Hypothyroidism												
No	15	12.3	38	97.4	77	92.8	.302	21	95.5	94	94.0	.791
Yes	32	26.2	1	2.6	6	7.2		1	4.5	6	6.0	
Encephilitis												
No	49	40.2	39	100.0	83	100.0		22	100.0	100	100.0	
Yes	26	21.3	0	0.0	0	0.0		0	0.0	0	0.0	
Smoker												
No	39	32.0	26	66.7	58	69.9	.721	15	68.2	69	69.0	.904
Yes	83	68.0	13	33.3	25	30.1		7	31.8	31	31.0	
Flu like symptoms												
No	22	18.0	8	20.5	9	10.8	.150	4	18.2	13	13.0	.525
Yes	100	82.0	31	79.5	74	89.2		18	81.8	87	87.0	

The demographic, clinical, and serological data of participants, including age, gender, blood type, Rh factor, comorbidities (diabetes mellitus, hypertension, asthma, hypothyroidism, encephalitis), smoking status, and symptom severity during infection. Antibody presence was analyzed using both qualitative and quantitative measures. Chi-square analyses were performed to assess the influence of patient characteristics on seropositivity.

DM = diabetes mellitus, HTN = hypertension, SARS-CoV-2 = severe acute respiratory syndrome coronavirus 2.

### 2.3. Statistical analysis

Data were analyzed using the Statistical Package for Social Sciences software version 25. The data were divided into qualitative variables and quantitative variables. Qualitative variables were summarized as percentages, and Chi-square tests were applied to compare groups. A *P* value of < .05 was considered statistically significant.

## 3. Results

### 3.1. Demographics and seropositivity rates

A total of 122 participants (62 males, 60 females) from Northern Jordan were included. Among them, 67.68% retained antibodies at 12 months postinfection. Demographic characteristics and antibody positivity rates are presented in Table 1.

### 3.2. Antibody persistence over time

**Figure 2.**
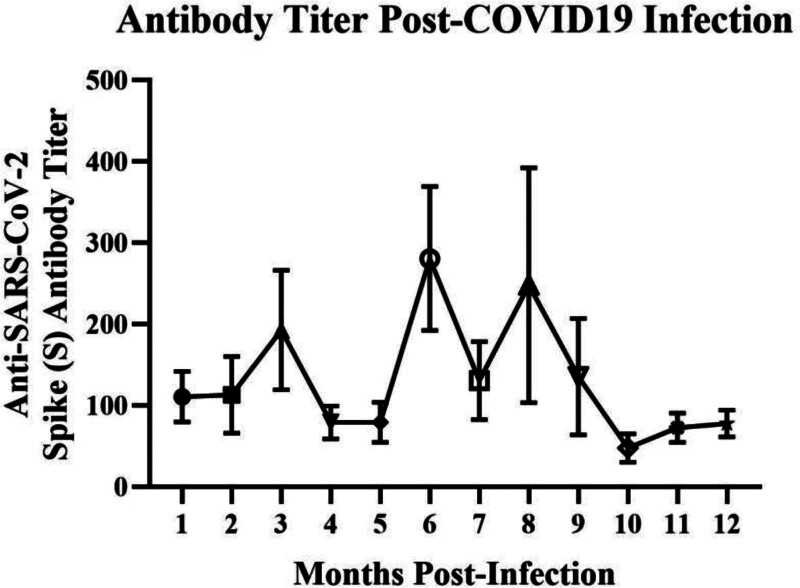
Longitudinal analysis of SARS‐CoV‐2 antibody titers (spike S protein) over 12 months post‐infection. The monthly detection of SARS‐CoV‐2 antibodies against the spike (S) protein in infected individuals. Serum samples were collected at different time points post‐infection and analyzed using the Elecsys Anti‐SARS‐CoV‐2 S assay (Roche Diagnostics GmbH, Mannheim). SARS-CoV-2 = severe acute respiratory syndrome coronavirus 2.

Seropositivity for both S and N proteins generally increased within the first 6 months postinfection; however, for S-specific antibodies, a transient dip was observed at months 4 and 5, followed by a subsequent increase at month 6, as shown in Figure 2 and Table 2. Despite this decline, antibody titers remained detectable in over 50% of patients even after 1 year of infection. Specifically, an average of 67.68% of patients continued to test positive for antibodies during the 10th, 11th, and 12th months. Notably, antibodies against the S protein demonstrated greater persistence in the serum, with consistently higher percentages of positive samples, compared to N antibodies.

**Table 2:** Monthly seropositivity rates for SARS-CoV-2 spike and nucleocapsid proteins.

Months post SARS-CoV-2 infection	1	2	3	4	5	6	7	8	9	10	11	12
Percentage of patients with positive SARS-CoV-2 antibody titer (S protein) (%)	89	89	100	100	91	100	70	75	67	38	100	60
Percentage of patients with positive SARS-CoV-2 antibody titer (N protein) (%)	70	78	100	85	91	78	60	50	56	42	58	50

The percentage of participants with detectable SARS-CoV-2 antibodies against spike (S) and nucleocapsid (N) proteins at different months postinfection. Serum samples were analyzed using the quantitative Elecsys Anti-SARS-CoV-2 S and the qualitative Elecsys Anti-SARS-CoV-2 assays, respectively (Roche Diagnostics GmbH). Percentages shown were calculated as the number of participants testing positive for antibodies at each month divided by the total number tested during that month. Qualitative variables were summarized as frequencies and percentages, and comparisons between groups were performed using chi-square tests. A *P* value of <.05 was considered statistically significant.

SARS-CoV-2 = severe acute respiratory syndrome coronavirus 2.

### 3.3. Influence of patient characteristics

Chi-square analyses were performed to assess the influence of patient characteristics on seropositivity. Blood group O was associated with the lowest seropositivity rates (*P* = .06). However, no significant associations were observed between seropositivity and other patient characteristics, including age, gender, comorbidities, smoking status, or symptom severity during the period of infection.

## 4. Discussion

The Ministry of Health in Jordan confirmed the first case of COVID-19 on March 2, 2020.^[16]^ Since then, over a million cases have been confirmed, representing approximately 10% of the Jordanian population. Given the scale of the outbreak, understanding the immune response to SARS-CoV-2 is crucial in guiding public health strategies, particularly in developing countries with limited healthcare resources.

This study investigated the duration of humoral immune response in COVID-19 patients by conducting a year-long kinetic analysis of antibody response in plasma samples from 122 unvaccinated volunteers. The influence of patient demographics and medical history was also assessed.

Our findings indicate that approximately 67.68% of plasma donors retained detectable antibody levels for at least 12 months postinfection. These results are consistent with the findings of Li et al, who reported seropositivity persistence in 70% of plasma samples 1 year after infection,^[17]^ as well as those of Yao et al, who concluded that the immune memory response persists for approximately a year.^[18]^

Importantly, the study found that antibodies against the S protein remained detectable for a longer duration compared to N antibodies. This observation aligns with the findings of Fenwick et al, who reported that humoral responses against nucleocapsid proteins declined more rapidly than those against the spike protein during the postinfection phase.^[19]^ These results suggest that the spike protein elicits a more robust and sustained immune response, which may have implications for vaccine design and durability of immunity.

Interestingly, the dip in antibody titers at months 4 and 5 followed by a rebound at month 6 suggests a biphasic pattern. Similar transient declines have been reported in other longitudinal studies. For instance, Seow et al demonstrated an early decline in neutralizing antibody titers within the first 3 months postinfection, followed by a stabilization phase, which was attributed to the waning of short-lived plasma cells and the subsequent contribution of long-lived plasma cells.^[20]^ A comparable biphasic pattern was also observed by Wajnberg et al who reported an initial decline in antibody titers over the first month postinfection followed by stabilization, consistent with the transition from short-lived plasmablast-derived antibodies to long-lived plasma cell production.^[21]^ Likewise, Gaebler et al observed that despite declining antibody levels, memory B cells continued to evolve and produced antibodies with increased potency and breadth several months after infection.^[22]^ Collectively, these studies suggest that the transient decline observed in our cohort may represent the natural contraction phase of short-lived plasma cells, followed by reactivation or maturation of long-lived memory B cells. Additionally, cohort size, individual variability in immune kinetics, or assay-related factors may have contributed to these fluctuations. Such findings reinforce that antibody dynamics are nonlinear and highlight the importance of longitudinal monitoring when interpreting antibody durability.

The analysis of patients’ characteristics revealed that individuals with blood group O were least likely to develop a humoral response to SARS-CoV-2. This finding is supported by studies from Bloch et al and Hayes et al, which found that more individuals with blood group B exhibited higher antibody titers compared to those with blood group O.^[23,24]^ The biological mechanisms underlying this observation remain unclear, but previous research suggests that ABO blood group antigens may influence susceptibility to viral infections. This finding may be relevant to donor recruitment in convalescent plasma therapy and warrants further investigation.^[24]^ We also assessed whether age, gender, preexisting medical conditions or comorbidities (including diabetes mellitus, hypertension, asthma, hypothyroidism, and encephalitis), smoking status, or symptom severity influence the persistence of SARS-CoV-2 antibodies. The rationale for including these comorbidities was based on prior studies suggesting that chronic illnesses may alter immune responses, potentially affecting the duration of postinfection immunity.^[25]^ However, in our study, no statistically significant associations were found between seropositivity and age, gender, comorbidities, smoking status, or symptom severity. While some studies, such as those conducted by Ciarambino et al and Yang et al, suggest that females and younger individuals may develop stronger antibody responses,^[26,27]^ our study did not observe the differences. The lack of significance could be attributed to the relatively small sample size, highlighting the need for larger cohort studies to confirm these findings.

Hospitalization and disease severity have been linked to immune response in prior research. Yu et al reported that hospitalized patients with medical comorbidities exhibited a higher magnitude of immune response compared to those with mild or asymptomatic infection.^[28]^ Similarly, Ferrara et al found that vaccine-induced antibody titers declined more rapidly among smokers than nonsmokers.^[29]^ However, our study did not establish a significant association between the humoral immune response to COVID-19 and medical comorbidities or smoking status. Regarding the association of the immune response with symptomatic infection, as noted by Marchi et al, only a few asymptomatic individuals develop detectable antibody levels. This underscores the importance of vaccination for those previously infected.^[30]^

These findings have several clinical implications. First, the prolonged persistence of S protein antibodies suggests that individuals who have recovered from COVID-19 may retain some degree of immunity for at least 1 year. This may reduce the urgency for booster vaccinations in previously infected individuals, particularly in regions where vaccine supply is limited. Moreover, identifying individuals with existing immunity is crucial to prioritizing vaccination efforts, ensuring that high-risk or nonimmune individuals receive doses first. This is particularly important for healthcare workers, as their placement within hospital settings should consider their immune status. Those with a confirmed past infection and likely immunity may be more safely assigned to high-risk zones, while uninfected or nonimmune staff should be prioritized for vaccination and potentially placed in lower-risk areas until protection is ensured.

From a public health perspective, the study reinforces the importance of continued serological surveillance to monitor population immunity levels. The observed differences in antibody persistence between S and N proteins further support the prioritization of S-based vaccines, which may offer longer-lasting protection. Moreover, the lower seropositivity rates in individuals with blood group O raise questions about potential variations in immune responses that warrant further exploration.

Finally, the findings of this study remain highly relevant even after the official end of the pandemic, as new variants of SARS-CoV-2 and related coronaviruses may emerge. Therefore, this study provides critical insights and serves as a valuable foundation for effectively responding to future outbreaks.

## 5. Conclusion

In conclusion, this study provides valuable insights into the long-term humoral immune response following natural SARS-CoV-2 infection. While antibody titers decline over time, a significant proportion of individuals, approximately 70%, maintain detectable antibody levels for a year post diagnosis, particularly for S protein antibodies. These findings support the notion that neutralizing antibodies can contribute to prolonged immunity, potentially preventing reinfection and enhancing vaccine efficacy.

Moreover, our results suggest that vaccination may not be immediately necessary for individuals who were previously infected within the past year, particularly in regions with limited vaccination resources. This is further supported by the possibility that humoral protection provided by immunization may persist beyond the measurable antibody response. However, further studies with larger sample sizes are needed to refine these conclusions and inform public health policies effectively.

## 6. Limitations

The study has some limitations, including a small sample size and the recruitment of participants from within a hospital setting, which may not fully represent the general population. To draw more precise conclusions, further research should involve a larger sample and a more diverse cohort with proper participant matching. Additionally, recruitment was limited to the period before the widespread rollout of vaccination in Jordan and prior to the emergence of new SARS-CoV-2 variants. While this limited the number of participants available, it also ensured that the study population reflected infections with the early circulating lineages, thereby reducing the likelihood of reinfection influencing the results. Finally, although we attempted to minimize this risk by recruiting before the emergence of the variant, the possibility of undetected reinfections cannot be completely excluded.

## Author contributions

**Conceptualization:** Nasr Alrabadi, Haneen Obeidat, Razan Haddad, Osama Alzoubi, Daher Al-rabadi, Saied Jaradat, Maryam K. El-zubi, Rania Harati, Karem H. Alzoubi, Ibrahim Al-faouri.

**Data curation:** Nasr Alrabadi, Haneen Obeidat, Razan Haddad, Osama Alzoubi, Daher Al-rabadi, Saied Jaradat, Maryam K. El-zubi, Rania Harati, Karem H. Alzoubi, Ibrahim Al-faouri.

**Formal analysis:** Nasr Alrabadi, Osama Alzoubi.

**Investigation:** Nasr Alrabadi, Haneen Obeidat, Razan Haddad, Osama Alzoubi, Daher Al-rabadi, Saied Jaradat, Maryam K. El-zubi, Rania Harati, Karem H. Alzoubi, Ibrahim Al-faouri.

**Methodology:** Nasr Alrabadi, Haneen Obeidat, Osama Alzoubi, Maryam K. El-zubi.

**Project administration:** Nasr Alrabadi, Razan Haddad, Saied Jaradat, Karem H. Alzoubi, Ibrahim Al-faouri.

**Resources:** Nasr Alrabadi, Razan Haddad, Saied Jaradat, Karem H. Alzoubi.

**Software:** Osama Alzoubi.

**Supervision:** Daher Al-rabadi, Ibrahim Al-faouri.

**Validation:** Nasr Alrabadi, Haneen Obeidat, Osama Alzoubi, Daher Al-rabadi.

**Visualization:** Nasr Alrabadi, Osama Alzoubi, Maryam K. El-zubi, Rania Harati, Ibrahim Al-faouri.

**Writing – original draft:** Nasr Alrabadi, Haneen Obeidat, Razan Haddad, Osama Alzoubi, Daher Al-rabadi, Saied Jaradat, Maryam K. El-zubi, Rania Harati, Karem H. Alzoubi, Ibrahim Al-faouri.

**Writing – review & editing:** Nasr Alrabadi, Razan Haddad, Karem H. Alzoubi.
